# A Miniaturized 3D-Printed Quartz-Enhanced Photoacoustic Spectroscopy Sensor for Methane Detection with a High-Power Diode Laser

**DOI:** 10.3390/s23084034

**Published:** 2023-04-17

**Authors:** Yanjun Chen, Tiantian Liang, Shunda Qiao, Yufei Ma

**Affiliations:** National Key Laboratory of Science and Technology on Tunable Laser, Harbin Institute of Technology, Harbin 150001, China

**Keywords:** quartz-enhanced photoacoustic spectroscopy (QEPAS), trace gas sensing, methane (CH_4_) detection, 3D-printing, high-power diode laser

## Abstract

In this invited paper, a highly sensitive methane (CH_4_) trace gas sensor based on quartz-enhanced photoacoustic spectroscopy (QEPAS) technique using a high-power diode laser and a miniaturized 3D-printed acoustic detection unit (ADU) is demonstrated for the first time. A high-power diode laser emitting at 6057.10 cm^−1^ (1650.96 nm), with the optical power up to 38 mW, was selected as the excitation source to provide a strong excitation. A 3D-printed ADU, including the optical and photoacoustic detection elements, had a dimension of 42 mm, 27 mm, and 8 mm in length, width, and height, respectively. The total weight of this 3D-printed ADU, including all elements, was 6 g. A quartz tuning fork (QTF) with a resonant frequency and Q factor of 32.749 kHz and 10,598, respectively, was used as an acoustic transducer. The performance of the high-power diode laser-based CH_4_–QEPAS sensor, with 3D-printed ADU, was investigated in detail. The optimum laser wavelength modulation depth was found to be 0.302 cm^−1^. The concentration response of this CH_4_–QEPAS sensor was researched when the CH_4_ gas sample, with different concentration samples, was adopted. The obtained results showed that this CH_4_–QEPAS sensor had an outstanding linear concentration response. The minimum detection limit (MDL) was found to be 14.93 ppm. The normalized noise equivalent absorption (NNEA) coefficient was obtained as 2.20 × 10^−7^ cm^−1^W/Hz^−1/2^. A highly sensitive CH_4_–QEPAS sensor, with a small volume and light weight of ADU, is advantageous for the real applications. It can be portable and carried on some platforms, such as an unmanned aerial vehicle (UAV) and a balloon.

## 1. Introduction

Methane (CH_4_), the simplest organic matter and the hydrocarbon with the least carbon and the most hydrogen, is one of the most potent greenhouse gases [1]. Since the industrial revolution, the concentration of CH_4_ has reached the level of ~2 ppm in the atmosphere. Although the concentration of CH_4_ in the atmosphere is small, its greenhouse effect is about 20 times that of carbon dioxide (CO_2_). Because of the severe influence of CH_4_ on climatic change and global temperatures rising, it is urgent to monitor the concentration of CH_4_ as well as find its main source. Meanwhile, CH_4_ is a flammable and explosive gas that can produce an explosion when the CH_4_ concentration is higher than 4% [2]. What is worrying is that CH_4_ with a high concentration can be found in mining and sewage pipelines, which brings huge security risks in cave mining and daily life. It is mandatory to detect CH_4_ sensitively for environmental monitoring and safety in industrial production and human life [3].

At present, the methods of gas monitoring are mainly divided into two types: spectroscopy and non-spectroscopy. Non-spectral measurement methods mainly include chromatographic analysis, mass spectrometry analysis, semiconductor sensors, insulator sensors, electrochemical sensors, and so on. Among non-spectral measurement methods, chromatographic analysis and mass spectrometry analysis are highly sensitivity. In particular, gas chromatography technology has a high degree of confidence in the measurement results thanks to the detection of pure gas samples via separation. However, the defects of gas chromatography technology are that the response time is slow and it is impossible to track real-time changes in gas concentration. In gas chromatography technology, a single sampling point can only detect the measurement data in a certain place in the measuring field and can’t accurately give the true value of the gas concentration in the entire measurement field. In addition, semiconductor and electrochemical gas sensors mostly have the shortcomings of low sensitivity, poor stability, and susceptibility to ambient influences. For example, semiconductor sensors based on metal oxide film need high power consumption to keep the metal oxide at the 100–500 °C temperature range that makes the sensors sensible to variations in temperature and humidity. On the contrary, optical measurement is an advanced technique [4,5,6,7,8,9]. Especially, the spectroscopic approach is to measure the parameters related to the spectrum of the targeted gas for inverting the concentration and composition information of the gas. It becomes an ideal tool for gas detection with its characteristics of large range, multi-component, continuous operation, and strong robustness [10,11,12].

Gas-sensing technologies based on laser absorption spectroscopy have lots of merits, such as high sensitivity, rapid response time, and a high selectivity [13,14,15,16,17]. These techniques can be divided into three groups of (1) Direct absorption, such as tunable diode laser absorption spectroscopy (TDLAS) [18,19]; (2) Cavity-enhanced absorption, such as cavity-enhanced absorption spectroscopy (CEAS) [20]; (3) Indirect absorption, such as photoacoustic spectroscopy (PAS) and light-induced thermoelastic spectroscopy (LITES) [21,22]. Compared with TDLAS and CEAS, PAS is a zero-background technique and has no excitation wavelength limit [23,24,25]. Therefore, it is widely used in the gas-sensing field. In PAS, gas samples absorb energy of the modulated laser. Then, the absorbed energy is translated into heat energy by non-radiative transitions such as inelastic collisions among the surrounding molecules, resulting in raising local temperature and generating acoustic waves. The intensity of acoustic waves is relevant to the gas concentration and can be detected using a sensitive acoustic wave detector. PAS is in a more advantageous position on account of wide wavelength responses, small volume, and a wide dynamic range [26,27,28].

The traditional PAS is based on microphone detection. However, the low resonance frequency and low Q-factor of microphone confine its detection capabilities to a low degree in the PAS sensor system. Further, the microphone-based PAS sensor usually has a large size of photoacoustic cells [29]. In order to meliorate the property of PAS technique in the mass, a method first came up in the year of 2002 by replacing microphones with a quartz tuning fork (QTF) [30], which is named as quartz-enhanced photoacoustic spectroscopy (QEPAS).

A QTF is made of quartz material with tuning fork structural characteristics. A commercial QTF used in QEPAS has geometric dimensions of 6 mm in length, 1.4 mm in width, and 0.25 mm in thickness, respectively. Each arm length is 3.8 mm; the width is 0.6 mm, and the gap between the two arms is 0.3 mm. A QTF is an oscillating device made using the piezoelectric effect of quartz crystals. Piezoelectric effects include positive piezoelectric effects and inverse piezoelectric effects. When an external force is applied to some crystals in a certain direction, many heterologous polarized charges appear on two opposite surfaces. When the external force is removed, the two surfaces return to an uncharged state, and this polarization phenomenon caused only by strain or stress is called the positive piezoelectric effect. Conversely, mechanical distortion occurs when an electric field is applied to the electrodes on the crystal surface, a phenomenon known as the inverse piezoelectric effect. Only the symmetrical vibration of the tuning fork can produce an effective piezoelectric signal. The sound source must be close to the center of the gap between the two forks of the tuning fork to cause the QTF to vibrate symmetrically. In order to effectively excite the photoacoustic signal at this position, the excitation beam should pass through the gap between forks of the QTF, allowing gas absorption of the infrared laser to occur in that position. Since the wavelength of the external noise is much larger relative to the fork gap, the sound waves generated from the distant sound source causes both forks of the QTF to move in the same direction, which does not excite an effective piezoelectric response.

**Figure 1:**
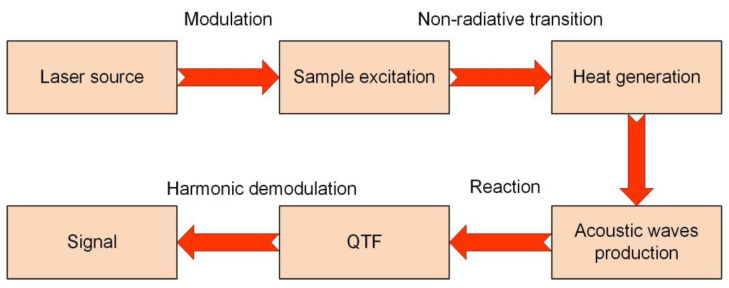
The schematic diagram of QEPAS.

The principle of QEPAS is shown in Figure 1. The laser source is modulated at half of the resonance frequency of the QTF. Owing to the piezoelectricity of quartz, the QTF acting as a transducer detects the intensity of the acoustic wave, which matches up with double micro-resonator tubes (mR) to strengthen the acoustic wave and signal of QEPAS. The advantages of the piezoelectric QTF, such as small size, low price, high Q, and strong anti-interference, are fully utilized in QEPAS [31,32,33]. Hence, the QEPAS sensor has the merits of tiny size, high sensitivity, and low cost. Many kinds of QEPAS-based sensors have been invented in the past few years so as to meet the detection requirements of different trace gases [34,35,36,37].

The signal level *S* of QEPAS sensor is determined by the Equation (1) [38].

(1) $$S\propto \frac{\alpha PQ}{f_{0}}$$

where *f*_0_ is the resonant frequency of QTF, *α* is the absorption coefficient of the sample, *P* is the optical output power, *Q* is the Q-factor of QTF. It is obviously obtained that the signal is inversely proportion to the resonance frequency. The reason why a drop in resonant frequency increases the signal is that the energy accumulation time will increase. As a result of using a QTF with a lower resonant frequency, the signal of QEPAS sensor can be effectively improved. From 2013, custom QTFs with different resonant frequency has been reported [39]. But the custom QTFs suffer from the high cost and is not beneficial to practical applications. In addition, from Equation (1), it can be derived that an excitation source with high output power is advantageous to increase the signal level. When a laser source with a higher optical power is employed in the QEPAS sensor, a stronger acoustic wave comes into being as a consequence of more excited gas molecules. However, the usually used single mode diode laser in QEPAS is less than twenty milliwatt (mW) in the near infrared region, which limits the sensor performance.

In the traditional QEPAS sensor, some dispersing elements such as optical and photoacoustic detection elements are usually used for detection. These independent elements such as optical lens and collimator weaken the system stability. Furthermore, machining process is widely adopted to produce photoacoustic unit. Such unit often suffers the drawbacks of big volume and heavy weight. In Ref. [40], the dimension of detection unit fabricated from machining were 50 mm, 30 mm and 21 mm in length, width and height respectively and the entire weight was 71 g. Assembling all parts is so tough and complex that it costs lots of time.

In order to play a great role in practical applications, a QEPAS sensor should be portable and lilliputian in terms of size and weight. Usually, the laser beam propagates and transforms with the help of a group of block-shaped lenses. As a result, it leads to reducing the stability of configurations and large sizes, hence the performance and practical application of the sensor are limited. The 3D printing technique does not require multi-machining process and traditional machine tools. It offers many merits, such as reduction in processing time and procedure, high integration as well as stability. A 3D-printed microresonator is reported in Ref. [41]. In this invited paper, a highly sensitive CH_4_ trace gas sensor based on QEPAS method using a high-power diode laser and a miniaturized 3D-printed acoustic detection unit (ADU) is demonstrated for the first time. A distributed feedback (DFB) diode laser emitting at 6057.10 cm^−1^ (1650.96 nm) with the optical power up to 38 mW was served as the excitation source to provide a strong excitation. Compare with the traditional QEPAS sensor fabricated from machining, by using a 3D-printed ADU, the photoacoustic detection and optical transmission elements are more compact and stable to avoid hassle of assembly of units. The performance of high-power diode laser based CH_4_-QEPAS sensor with 3D-printed ADU was investigated in details.

## 2. Experimental System

### 2.1. CH_4_ Absorption Line Selection

**Figure 2:**
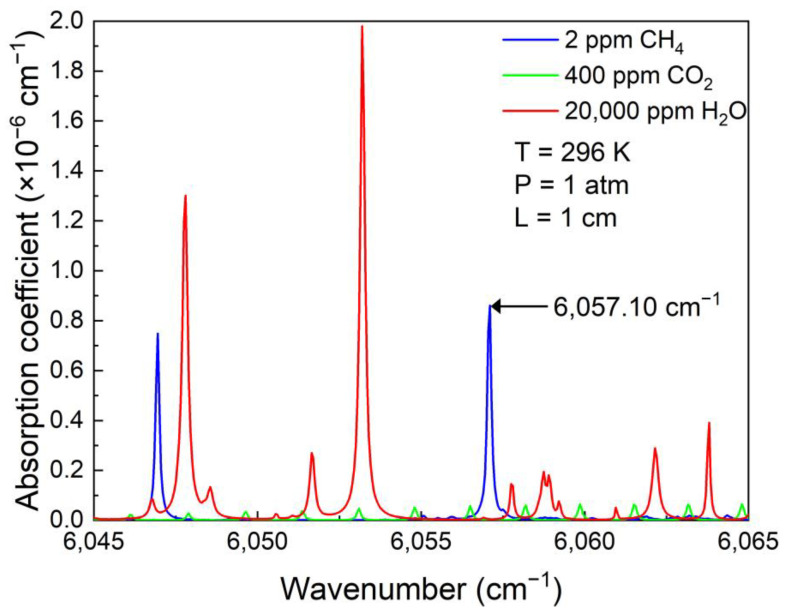
Absorption lines of CH_4_, H_2_O and CO_2_ at the conditions of 1 atm pressure, 296 K temperature and 1 cm optical absorption length using the database of HITRAN 2016.

The fundamental vibrational-rotational absorption band of CH_4_ is located at 3.3 μm. Although this band has the strongest absorption strength, it can only access by the interband cascade laser (ICL) with GaSb-AlGaInAsSb material series, which suffers a high cost. Diode lasers with emission wavelength in the near infrared, especially in the wavelength range short than 2 μm are mature and can be coupled with fiber. The output wavelength of the laser is selected according to the target absorption line of the gas molecule, which usually follows the following three principles: (1) strong excitation intensity; (2) separation from other background gas lines and no interference term and (3) availability of laser light sources suitable for the wavelength. Therefore, in this paper, a diode laser with output wavelength of 1.6 μm was used to target the CH_4_ absorption lines. The simulation of absorption line of CH_4_ in this band based on HITRAN 2016 database is shown in Figure 2 on the conditions of a standard atmospheric pressure (1 atm) and room temperature of 296 K. It is apparently seen from Figure 2 that the absorption line at 6057.10 cm^−1^ (1650.96 nm) is one of the strongest CH_4_ absorption lines around 1650 nm. Furthermore, water vapor (H_2_O) and CO_2_ do not interfere with CH_4_ in this wavelength range.

### 2.2. DFB Diode Laser Output Characteristics

**Figure 3:**
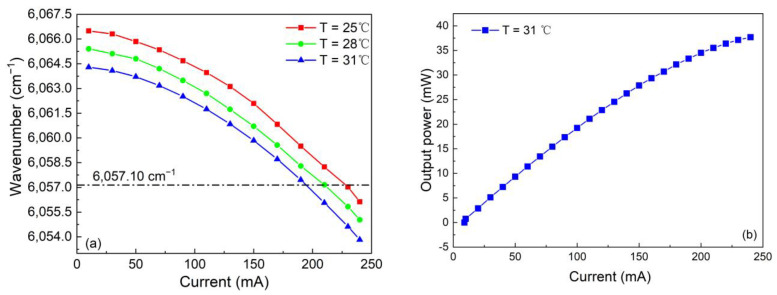

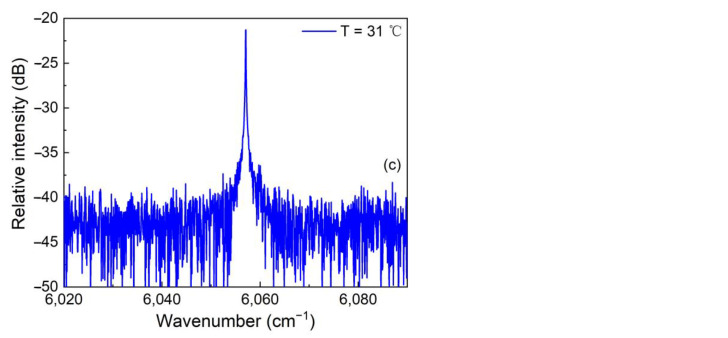
The output performance of the 6057.10 cm^−1^ CW-DFB laser. (**a**) The trend of laser output wavelength. (**b**) The laser output power. (**c**) The emission spectrum of the 6057.10 cm^−1^ CW-DFB laser.

The output performance of diode laser such as the wavelength is typically tuned by changing the temperature of thermoelectric control (TEC) or injection current to target the desired wavelength. The used DFB diode laser in this experiment is in continuous wave (CW) mode. The output performance of the CW-DFB diode laser was measured and is presented in Figure 3. As shown in Figure 3a, the desired wavelength of 6057.10 cm^−1^ was achieved when the injection current was 196 mA, 212 mA and 230 mA at the TEC temperature of 25 °C, 28 °C and 31 °C, respectively. The measured tuning coefficients for the temperature and current of this CW-DFB laser were −0.37 cm^−1^/mA and −0.066 cm^−1^/mA, respectively. In order to protect the diode laser from damage and prolong the lifespan, the TEC temperature of the diode laser was set to 31 °C so that the injection current is in the modest range. As depicted in Figure 3b, the maximum optical output power was 38 mW when the injection current was 240 mA. The emission spectrum of the laser was recorded and shown in Figure 3c. We can find that the side-mode suppression ratio of this CW-DFB diode laser is higher than 25 dB, which indicated it had an excellent ability in the single mode laser output.

### 2.3. 3D-Printed Acoustic Detection Unit

**Figure 4:**
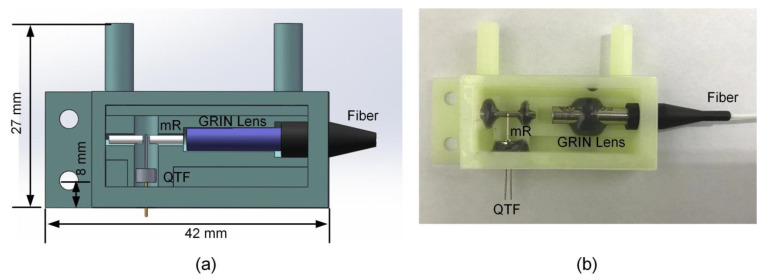
Schematic diagram of 3D-printed ADU: (**a**) design pattern; (**b**) assembled ADU.

3D-printing technique has the merits of high level of integration, high production efficiency, and low cost. For the sake of improving the size and stability of traditional QEPAS sensor fabricated from machining, 3D-printing was introduced to produce a ADU. An UV-curable resin was employed as the work material. The designed mode of ADU is shown in Figure 4a. It has a dimension of 42 mm, 27 mm and 8 mm in length, width and height, respectively. It contained a QTF and a pair of mRs. Furthermore, a fiber coupled GRIN lens was also incorporated into this ADU for laser transmission and collimation. The 3D-printed ADU was sealed by using quartz glass windows. The assembled ADU is shown in Figure 4b. The gross weight of this 3D-printed ADU including all elements was 6 g.

### 2.4. QEPAS Sensor Configuration

**Figure 5:**
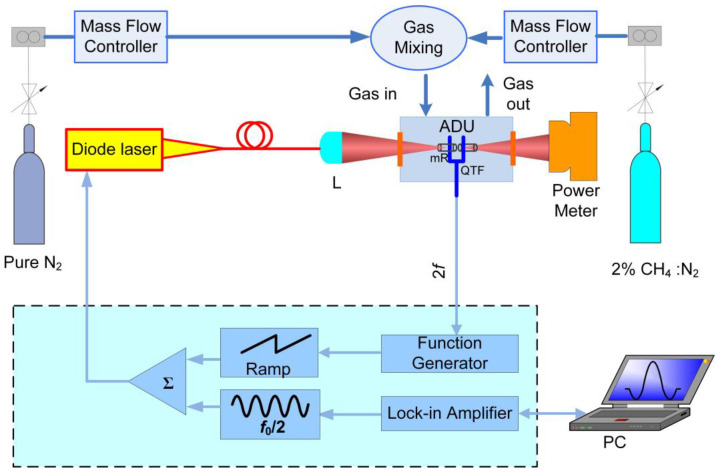
Schematic diagram of CH_4_-QEPAS sensor with 3D-printed ADU.

The schematic diagram of QEPAS sensor is shown in Figure 5. The output beam of this CW-DFB diode laser was collimated by a fiber coupled GRIN lens (L). Subsequently, the beam passed through the ADU, where a QTF and mR were placed. The mR was constituted by two steel tubes with length in the scope of *λ*_s_/4 to *λ*_s_/2, where *λ*_s_ is the wavelength of the generated acoustic wave. QTF with the resonant frequency (*f*_0_) of 32.768 kHz was adopted as the acoustic wave transducer. Therefore, the length of mR was selected as 5 mm in this investigation. After passing through the ADU, the laser beam was shot into a power meter to monitor the change of optical alignment. Wavelength modulation with harmonic detection strategy was used in this experiment to simplify the data analysis and suppresses background noise. A sawtooth wave with low frequency generated from a function generator was adopted to scan across the CH_4_ 6057.10 cm^−1^ absorption line. A sine wave with high frequency (*f = f*_0_/2) produced from a lock-in amplifier was used to modulate the wavelength of the CW-DFB diode laser. Two mass flow controllers consisting of a flowmeter controller and a needle valve were employed to set the flow rate and mixing ratio between pure nitrogen (N_2_) and CH_4_:N_2_ mixture with 2% CH_4_ concentration.

## 3. Experimental Results and Discussions

**Figure 6:**
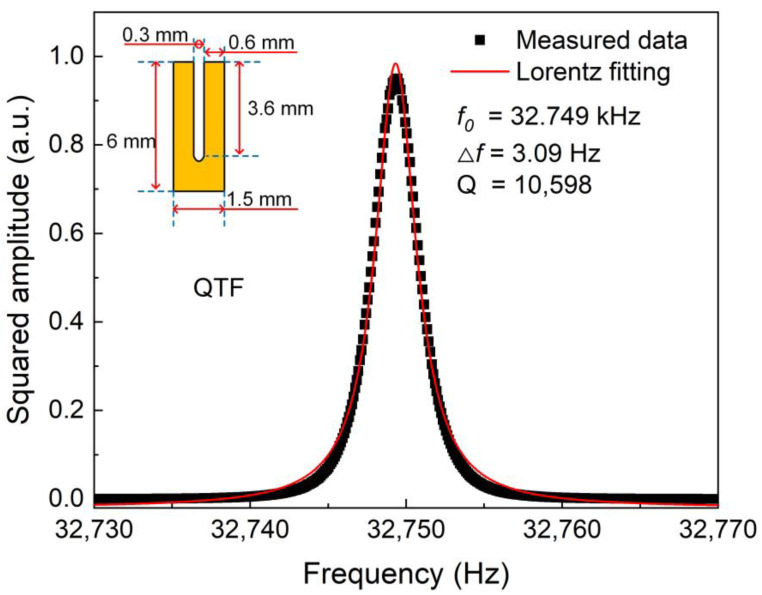
Frequency response of the used QTF.

The resonant frequency of QTF is affected by its physical dimension. The schematic size of the used QTF is shown in the insert of Figure 6. The length, width and depth of the QTF’s prong were 3.6 mm, 0.6 mm and 0.25 mm, respectively. The gap between the two prongs was larger than 0.8 mm. There are two methods of determining the resonant frequency of QTF, namely electrical excitation and optical excitation. The frequency curve obtained by the optical excitation method is more symmetrical than the achieved result from the electrical excitation. In this paper, the resonant frequency of the used QTF was measured by using the optical excitation method. In the measurement, the excitation frequency of the CW-DBF diode laser was scanned from 32.73 Hz to 32.77 Hz. The obtained result is shown in Figure 6. After using Lorentz fitting, the peak response (*f*_0_) and bandwidth (△*f*) of the resonant frequency were found to be 32.749 kHz and 3.17 Hz. According to the expression *Q* = *f*_0_/△*f*, the value of *Q* was determined to be 10,598.

**Figure 7:**
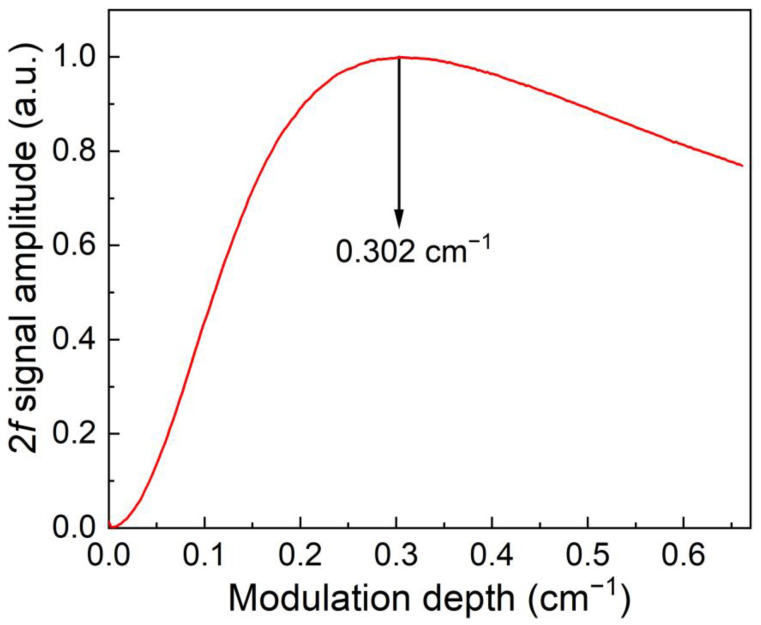
The 2*f* signal versus diode laser wavelength modulation depth for CH_4_-QEPAS sensor with 3D-printed ADU.

Laser wavelength modulation depth is an important parameter, which has a significant impact on the QEPAS sensor performance. The function between the 2*f* signal level and diode laser wavelength modulation depth was investigated and depicted in Figure 7. It can be found that firstly the signal level increased with the modulation depth. The signal reached the maximum when the laser wavelength modulation depth was 0.302 cm^−1^. After that, the 2*f* signal did not increased obviously. Therefore, in the following investigations, the optimum value of 0.302 cm^−1^ was adopted.

**Figure 8:**
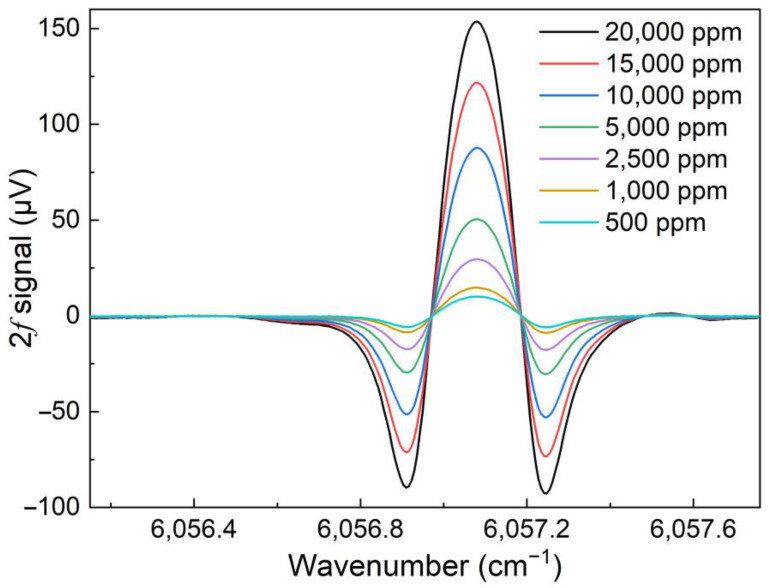
2*f* signal for CH_4_-QEPAS sensor with different CH_4_ concentrations.

**Figure 9:**
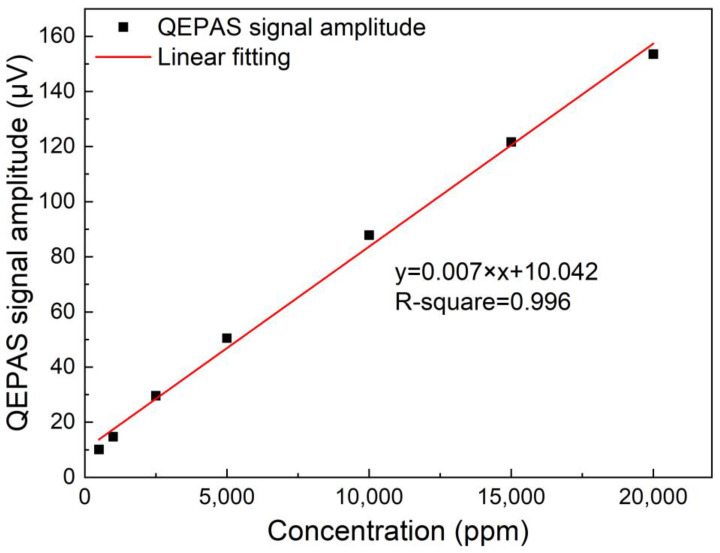
Concentration response of CH_4_-QEPAS sensor with 3D-printed ADU.

Through controlling the flow rate of two bottles of 2% CH_4_:N_2_ mixture and pure N_2_ using two flow controllers, the 2% CH_4_:N_2_ mixture was diluted to produce different CH_4_ concentration levels. The high-power diode laser based CH_4_-QEPAS sensor performance was investigated when CH_4_ gas sample with different concentration was adopted. The 2*f* signal waveforms for CH_4_ with concentration ranging from 500 ppm to 20,000 ppm is depicted in Figure 8. The signal amplitude for the high-power diode laser based CH_4_-QEPAS sensor with 3D-printed ADU with different concentrations was linear fitted. The obtained results are shown in Figure 9. It can be seen that the R-square for the linear fitting is 0.99, so that the CH_4_-QEPAS sensor had an excellent linear CH_4_ concentration response.

**Figure 10:**
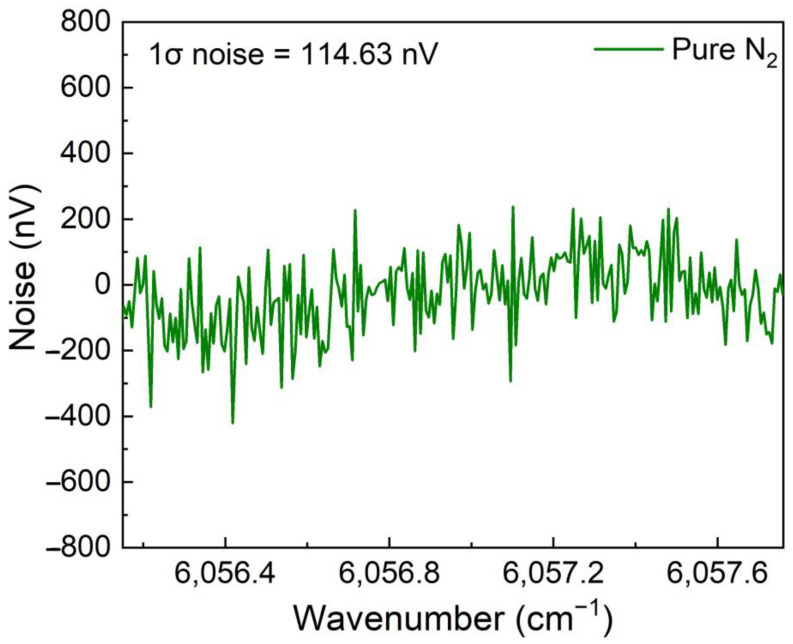
Noise level of CH_4_-QEPAS sensor with 3D-printed ADU.

The noise level of this high-power diode laser based CH_4_-QEPAS sensor was investigated when pure N_2_ was flowed into the ADU. The noise data was recorded and shown in Figure 10. The standard deviation (1σ noise) was found to be 114.63 nV. Therefore, the minimum detection limit (MDL) was calculated to be 14.93 ppm based on the equation MDL = C/SNR, where C is the measured CH_4_ concentration, SNR is the signal to noise ratio for the measurement.

The normalized noise equivalent absorption coefficient (NNEA) can also be used to assess the sensitivity of an optical sensor. The definition of NNEA is shown in Equation (2):

(2) $$\mathrm{NNEA}=\frac{\alpha P}{\sqrt{\Delta f}}$$

where *α* is the absorption coefficient of the selected absorption line, *P* is the emission optical power, and Δ*f* is the detection bandwidth of the used lock-in amplifier. The normalized noise equivalent absorption (NNEA) coefficient was achieved to be 2.20 × 10^−7^ cm^−1^W/Hz^−1/2^ for this CH_4_-QEPAS sensor. For comparison, the performance of CH_4_-QEPAS sensor using traditional machining and 3D-printed ADU is listed in Table 1. It can be seen that 3D-printed CH_4_-QEPAS sensor has a better performance.

## 4. Conclusions

In summary, a highly sensitive CH_4_ trace gas sensor based on QEPAS method using a high-power diode laser and a miniaturized 3D-printed ADU is demonstrated for the first time. A CW-DFB diode laser emitting at 1650.96 nm with the optical emission power up to 38 mW was served as the excitation source to provide a strong excitation. A 3D-printed ADU including the photoacoustic detection and optical transmission elements is more compact and stable when compared with the traditional unit made by machining. The ADU had a length of 42 mm, width of 27 mm and height of 8 mm. The gross weight of this 3D-printed ADU including all elements was 6 g. A QTF with resonant frequency and Q factor of 32.749 kHz and 10,598, respectively, was used as acoustic detector. The performance of high-power diode laser based CH_4_-QEPAS sensor with 3D-printed ADU was investigated in detail. The optimum wavelength modulation depth for the diode laser was found to be 0.302 cm^−1^. The concentration response of this CH_4_-QEPAS sensor was investigated when CH_4_ gas sample with different concentration ranging from 500 ppm to 20,000 ppm was adopted. The achieved results showed that the CH_4_-QEPAS sensor had an excellent linear concentration response. The noise level of this high-power diode laser based CH_4_-QEPAS sensor was investigated when pure N_2_ was flowed into the ADU. It is found that this sensor had a noise level of 153.54 nV, which resulted in a MDL of 14.93 ppm. The NNEA was calculated as 2.20 × 10^−7^ cm^−1^W/Hz^−1/2^. The highly sensitive CH_4_-QEPAS sensor with a small volume and light weight of ADU is advantageous for the real applications. It can be portable and carried on some platforms such as unmanned aerial vehicle and balloon.

## Figures and Tables

**Table 1:** The comparison between traditional machining and 3D-printed ADU.

Method	MDL (ppm)	NNEA (cm^−1^W/Hz^−1/2^)	Ref.
Machining	15	7.26 × 10^−6^	[42]
3D-printed	14.93	2.20 × 10^−7^	This paper

## Data Availability

The data presented in this study are available on request from the corresponding author.
